# Concept of Placement of Fiber-Optic Sensor in Smart Energy Transport Cable under Tensile Loading

**DOI:** 10.3390/s22072444

**Published:** 2022-03-22

**Authors:** Monssef Drissi-Habti, Neginhal Abhijit, Manepalli Sriharsha, Valter Carvelli, Pierre-Jean Bonamy

**Affiliations:** 1 COSYS Department, Université Gustave Eiffel, Champs/Marne, F-77447 Marne-la-Valleée, France; abhijit.neghinal@gmail.com (N.A.); sriharsha.manepalli@gmail.com (M.S.); 2 Department A.B.C., Politecnico di Milano, Piazza Leonardo Da Vinci 32, 20133 Milan, Italy; valter.carvelli@politecnico.it; 3 MEDYSYS, 91400 Orsay, France; pj.bonamy@medysys.fr

**Keywords:** smart composite, strain, fiber optic sensor (FOS), cross-linked polyethylene (XLPE), multi-axial strains, smart energy transport cable, thermomechanical coupling

## Abstract

Due to the exponential growth in offshore renewable energies and structures such as floating offshore wind turbines and wave power converters, the research and engineering in this field is experiencing exceptional development. This emergence of offshore renewable energy requires power cables which are usually made up of copper to transport this energy ashore. These power cables are critical structures that must withstand harsh environmental conditions, handling, and shipping, at high seas which can cause copper wires to deform well above the limit of proportionality and consequently break. Copper, being an excellent electric conductor, has, however, very weak mechanical properties. If plasticity propagates inside copper not only will the mechanical properties be affected, but the electrical properties are also disrupted. Constantly monitoring such large-scale structures can be carried out by providing continuous strain using fiber-optic sensors (FOSs). The embedding of optical fibers within the cables (not within the phase) is practiced. Nevertheless, these optical fibers are first introduced into a cylinder of larger diameter than the optical fiber before this same fiber is embedded within the insulator surrounding the phases. Therefore, this type of embedding can in no way give a precise idea of the true deformation of the copper wires inside the phase. In this article, a set of numerical simulations are carried-out on a single phase (we are not yet working on the whole cable) with the aim of conceptualizing the placement of FOSs that will monitor strain and temperature within the conductor. It is well known that copper wire must never exceed temperatures above 90 °C, as this will result in shutdown of the whole system and therefore result in heavy maintenance, which would be a real catastrophe, economically speaking. This research explores the option of embedding sensors in several areas of the phase and how this can enable obtaining strain values that are representative of what really is happening in the conductor. It is, therefore, the primary objective of the current preliminary model to try to prove that the principle of embedding sensors in between copper wires can be envisaged, in particular to obtain an accurate idea about strain tensor of helical ones (multi-parameter strain sensing). The challenge is to ensure that they are not plastically deformed and hence able to transport electricity without exceeding or even becoming closer to 90 °C (fear of shutdown). The research solely focuses on mechanical aspects of the sensors. There are certainly some others, pertaining to sensors physics, instrumentation, and engineering, that are of prime importance, too. The upstream strategy of this research is to come up with a general concept that can be refined later by including, step by step, all the aspects listed above.

## 1. Introduction

Extensive research work has been devoted to offshore wind generation in recent years in aspects of both large-dimension wind blades and high-voltage electrical transport cables [1,2,3,4,5,6,7]. The key point of structural health monitoring (SHM) is the adequate technology selection and the most accurate placement of the necessary sensors for measurement of parameters that are directly responsible for the performance of the system. The health of the system is influenced by type of loading, magnitude of loading, the area where the cable is placed (for example, area where the cable is heavily deformed under bending), pre-existing anomalies in the installation surfaces, anchor drops, and shark attacks. The influences of individual events are manifested in terms of perturbations such as strains, damage, and/or cracks, which can lead to thermal gradients and high heat. Therefore, selection of appropriate sensors requires knowledge of the relationship between the events and their effects [8]. According to current market survey, the energy produced by offshore farms is financially costlier than the energy produced by fossil fuels. The difference becomes much smaller if we consider the side effects caused by the fossil fuels, such as global warming and environmental pollution. This difference could be improved by reducing the maintenance cost [9]. This can be made possible by considering several techniques, among which structural health monitoring (SHM) is one. In this decade, the offshore cable failures account for 80% of total financial losses and insurance claims. In the past 7 years, about 90 offshore cable failures have been reported with over EUR 350 million in insurance claims. According to [10], the repair costs of an offshore cable can be between EUR 0.7 million and EUR 1.5 million, which is very expensive.

**Figure 1:**
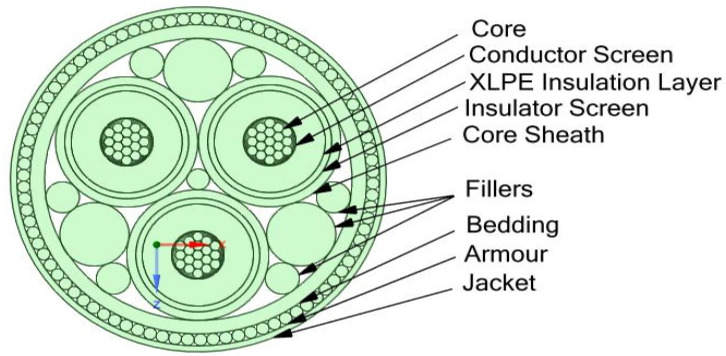
Cross-section of the cable.

**Figure 2:**
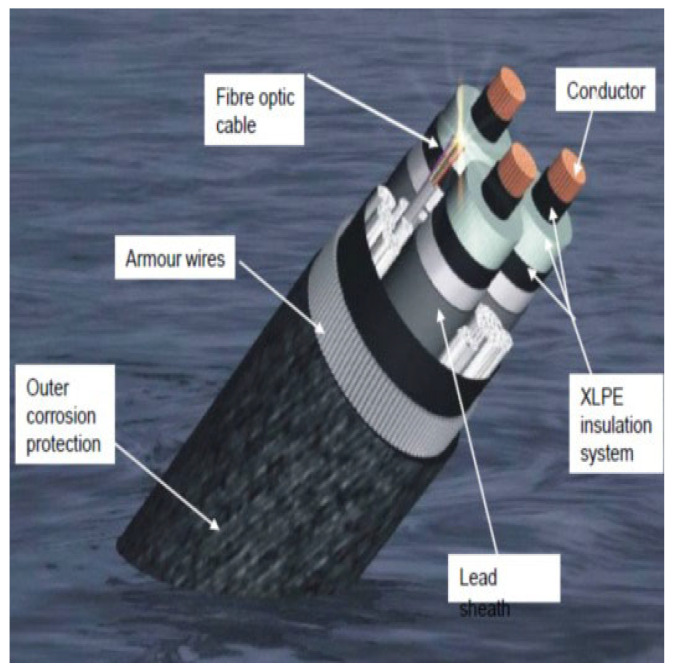
Electric transport cable for offshore farms [11].

The technique of embedding FOS can help monitor the cable (Figure 1 and Figure 2) [11]. There are a wide variety of applications of embedding fiber optic sensors in composite materials which include vibration measurements, cure process monitoring, temperature measurements, thermal expansion measurements, detection of delamination/debonding, three-dimensional strain measurements, thermal strain measurements, relative humidity measurements, and detection of cracking. For all these applications, the key requirement is to measure either strain or temperature or both parameters [12]. The different types of FOS reported for strain/temperature measurements in composite materials are fiber Bragg grating (FBG) sensors, interferometric fiber optic sensors, polarimetric sensors, fiber optic micro-bend sensors, distributed sensors (using techniques such as Rayleigh scattering, Raman scattering, and Brillouin scattering), and hybrid sensors.

The core of the power phase is made up of copper, which is a weak metal. If plasticity propagates inside copper wires, both their individual mechanical properties and their individual electrical properties are disrupted. Plasticity is the consequence of massive defects proliferation into crystal lattices of the metal that lower electrical and thermal conduction [13]. Looking at the generic structure of submarine power cables, there is no potential room for inserting a separate cable with FOS inside it for monitoring of the core. Nowadays, optical fibers which are used for communications are placed inside the cable in place of the fillers with their separate individual casing. Placing the FOS, to monitor the health of the core inside the filler, means placing the FOS far from the copper core, and this may result in inaccurate data due to major offset and presence of other materials, of which some might be in contact and some may not. Along with this, placing another cable for FOS adds up to more space inside the submarine cable. The increase in small space accounts for millions of funds when considered for thousands of kilometers of submarine power cables. Thus, this article suggests a solution of embedding FOS in between copper wires or within the insulator XLPE (cross-linked polyethylene). The placements strategy is balanced between two aspects; first, when placing fiber-optics within copper wires, sensors will be facing extensive sliding friction, which may be detrimental and leads to their failure. Second, embedding sensors within the insulator seems easy, but technically may lead to extensive and variable offset. This work is intended to numerically study these two strategies and extract the most feasible cases. It is worthwhile to note that this article is tackling the issues from a mechanical prospective, knowing that there are other aspects related to sensors physics and instrumentation that are not addressed.

Presently, XLPE is widely used in submarine cables due to its good electrical and thermomechanical behaviors and relatively low cost [14]. The maximum heat-resistant operating temperature of XLPE insulation is 90 °C and the overload temperature should be less than 105 °C [15] in order to avoid premature aging of the dielectric [16]. If one or more copper wires are broken and/or permanently deformed in the core, it leads to upshoot of the temperature of the core [7], and once the core rises above 90 °C, the whole system shuts down. In addition, high temperature will accelerate XLPE insulation aging and shorten cable service life [17]. On the contrary, if the operating temperature of the cable conductor is much lower than 90 °C, the operating efficiency of the cable will be reduced [18]. Therefore, conductor temperature will directly affect the aging of XLPE insulation and the operation of cable; thus, real-time cable temperature measurement is critical to the safe and steady operation of the XLPE power cable [19]. Monitoring of stress and temperature through embedded sensors is therefore mandatory. This is the main reason for this work.

Embedding of optical sensors within XPLE is quite easy to handle technically, whereas embedding between copper wires is technologically challenging. Indeed, either embedding along the central copper wire and/or helically-wound the same way as other copper wires can lead to extensive sliding/friction and eventually fiber-optic sensor failure. To avoid this, FOSs can be covered by friction-resistant fiber-optic coatings, whereas embedding within XPLE can underestimate the magnitude of thermal parameters inside the phases. The question raised in this article is whether the underestimate is constant or not, after the embedding location is shifted from copper to XPLE (at several distances). The objective of the current article is to check these points numerically, which will help further engineers to target the right cost-effective solutions. Until today, optical fibers are not embedded within the phase, but in the cables and, more precisely, in the insulator that surrounds the phases. Moreover, such optical fibers are not embedded to monitor wire plasticity (fine damage) as we wish to do. Indeed, these optical fibers are embedded into a cylindrical tube, with a diameter much greater than that of the optical fiber, and this difference in diameter in no way makes it possible to follow the true deformation of the copper wires. What we bring back as innovation consists of (i) the embedding of the optical fiber in the phase (and not in the cable), (ii) the embedding of the optical fibers between copper wires to monitor true wire strains, along the central wire in order to measure its deformation, (iii) around the helical wires in order to measure their 3D deformation as well, and (iv) in the insulator at different distances from the center of the phase, to check for the magnitude of the strain offset vs. strain measured at the central wire location. It is worthwhile to note that the article is concentrating on mechanical aspects pertaining to sensors embedding. The aspects linked to the type of sensors physics and associated engineering are too premature to address at this stage.

## 2. Geometric Model

We will be working on a single phase of the 35kV Nexans submarine power XLPE cable, 2XS2YRAA 18/30(36) kV. The conductor screen is insulated by a thick layer of XLPE (cross-linked polyethylene). The XLPE insulation is mostly recommended for usage in submarine cables as they perform well in both high and low temperatures [20]. At high temperature, the elastic modulus of XLPE decreases while the thermal expansion increases. XLPE has a high thermal expansion as its temperature increases from 25 °C to 105 °C, XLPE expands by 5% while the copper expands by less than 3% in the same temperature range [21]. The insulator is surrounded by a thin insulator screen made up of semi-conducting material (Table 1).

## 3. Mechanical Boundary Conditions

Submarine cables are designed in such a way that they can withstand all mechanical stress during manufacturing, transport, handling, installation, and operation. These stresses imposed on submarine cables are completely different from the factors which are affecting the underground cables. In case of inappropriate design and layouts, the cable is more prone to damages and results in high repair cost. Thus, such inappropriate designs are usually abandoned and replaced rather than being rectified. From the previous studies, the tensile and torsional loading cases were considered on a cable armor by using the energy method to create a balance equation [22]. The cable will generate a torsional force when the cable is subjected to axial tensile loading, and vice versa will be in the case of torsional loading, that is, it will generate an axial tensile force [23]. With the introduction to finite element method, we can effectively analyze tensile properties of the cable. There was an observation made that the tensile stiffness of the cable increases with increase in the radial stiffness up to a typical value and then drops sharply [24]. In this article, a pure tensile loading of a single phase of the cable was assumed, which is not reflecting the reality. The idea here is to have the basic outputs for further research. In previous research, such as [24,25], the primary loading considered is a pure tensile loading case. The tensile load usually occurs while the cables are being laid from the ship. There are three major components which contribute to the tension forces at the laying wheel [26]:

(i) Static weight of the cable between the ship and sea floor.
(ii) Residual bottom tension, which translates to an extra tension force at the laying wheel.
(iii) Dynamic forces when the laying wheel is moving up and down.

**Figure 3:**
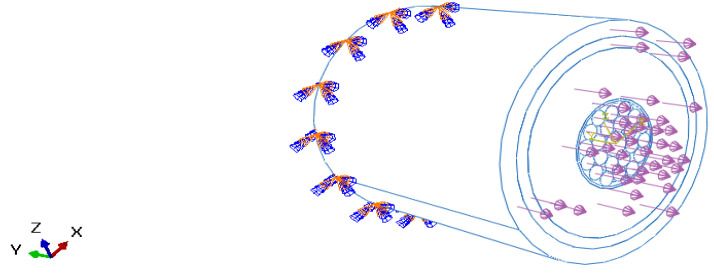
Pure tensile loading condition.

Tensile loading on one face of the phase was considered, as shown in Figure 3. The tensile loading is applied along the axial/longitudinal axis of the submarine phase with magnitude of 80 MPa. This stress value is considered according to the hydrostatic law assuming the phase is at a depth of 8000 m. The opposite end of the phase is constrained with all degrees of freedom fixed. Numerical simulations have been conducted assuming that copper wires are arranged in a concentric pattern in a three-layered configuration (1 + 8 + 14). These wires are helically wrapped around the central wire of the core. For the copper helical wires and the helical FOS (mentioned in another test case), the length:pitch ratio has been considered as 1:1 and the twist angle of the helical wires are modeled at 0 deg. The various parts of the model have been explained elsewhere (cf. references of the team, for example, Matine and Drissi-Habti). The contact assumed is linear. All simulations were conducted with a fiber-optic made of silica glass (density 2.4, Young’s modulus 72 GPa, and Poisson’s ratio 0.17) and acrylate coating (density 0.9, Young’s modulus 2.7 GPa, and Poisson’s ratio 0.35).

## 4. Thermal Boundary Condition

**Figure 4:**
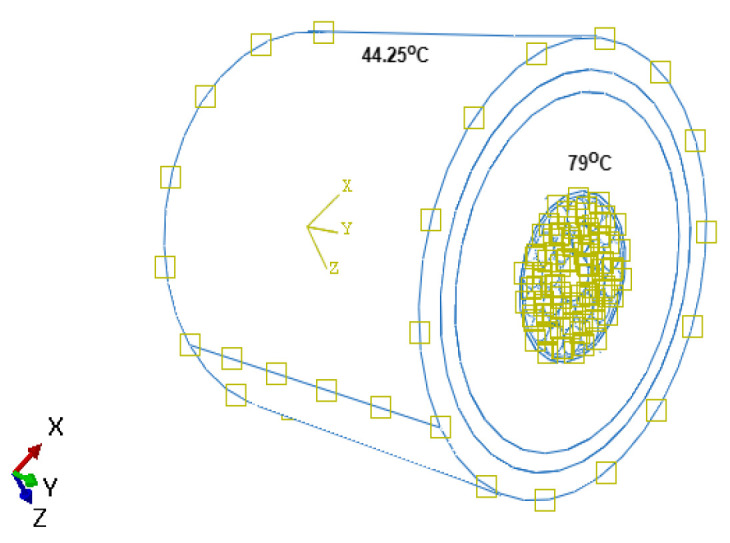
Thermal loading condition.

There are two common methods for calculating temperature and the ampacity of submarine cables. One is the equivalent thermal resistance method that is based on International Standard IEC 60287. This method has high efficiency and accuracy in calculating the temperature and the ampacity of the direct buried cables. It can satisfy the calculation of the load in the simple scenario. For instance, the IEC calculation cannot address the physical problems coupling with air convection, radiation, and heat transfer, and it will cause large errors in calculation of the multi-loop and complex environment. Another method is the numerical method, including the boundary element method [27], difference method, and finite element method [28]. The finite element method that is based on COMSOL can simulate actual working conditions and perform the coupling calculation of multiple physical fields [14]. The conventions of the IEC 60287 and working of cable at normal operation of the cable will be considered from [7]. In this paper [7], it has been considered that sea water is the surrounding media with an ambient temperature of 15 ${}^{\circ }$C. The thermal analysis of cables aims at computing the temperature rise inside the cables due to the heat generated inside the conductor. During the working of the submarine power cable, the heat is generated in the cable core (heat source) and is transported to the outside of the cable and disappears into the ambient water(heat sink). The transportation from the heat source to heat sink involves various different layers. The heat transfer is assumed to take place in a steady-state phase, i.e., the analysis will be independent of time (Figure 4). The transfer of heat is assumed to take place by conduction only, and the radiation effects are considered to be negligible. In addition, the material is considered isotropic, which means material properties are the same in all directions.

## 5. Placement of FOS

**Figure 5:**
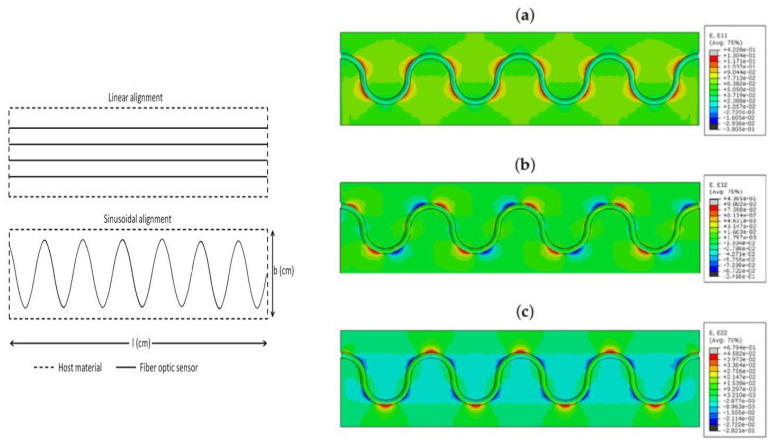
Illustration of linear and sinusoidal fiber FOS placement strategies: (**a**) E11 strain component; (**b**) E12 strain component; (**c**) E22 strain component (acrylate-coated FOS numerical model; the value observed in the host epoxy), sinusoidal placement. Multi-parameter strainsin linear, shear, and lateral directions) can be recorded on sinusoidal placement, while this is not shown when using straight-placed FOS.

In previous articles [1,2,3,4], simulations have been conducted to assess the placement of FOS linearly parallel to bonded joints versus a placement based on a sinusoidal fiber covering the largest part of bond joint surfaces and/or composite plies in the case of large-sized wind-blades for offshore farms Figure 5. It has been shown that a single sinusoidally-placed FOS can cover the largest part of the surface to be monitored, in comparison to a linearly-placed FOS concept that needs many parallel FOSs, which is costly. The monitoring that uses a sinusoidal-placed FOS comes up with multi-parameter strain recording, which is a significant advantage over linearly-placed FOS. The simulation conducted took care to refine the placement of FOS to the edge to avoid FOS slippage and sensibility reduction.

**Figure 6:**
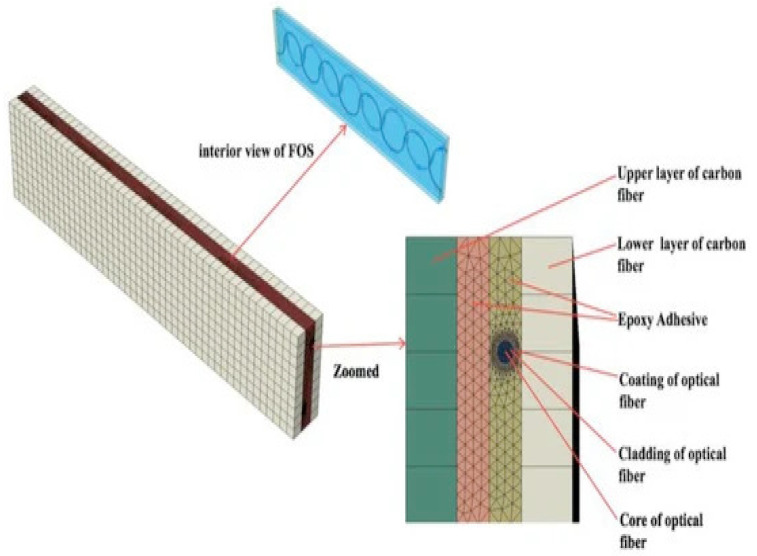
Orientation of dual-sinusoidal FOS.

**Figure 7:**
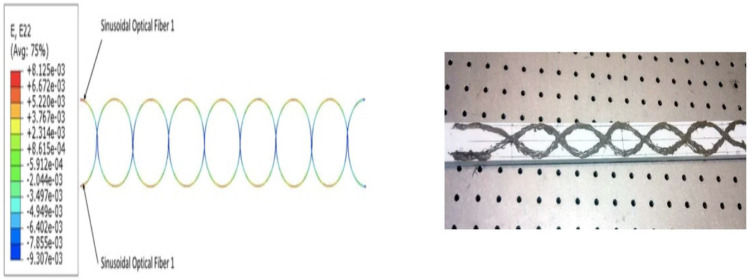
Dual-sinusoidal FOS placement embedded on a glass composite specimen along with strain measurements showing dual-sinusoidal FOS placement (placement in dual-sinusoidal mode, in phase opposition, provides coverage complementary to that of a single sinusoidal fiber) and a view of the specimen that was used for experimental validation.

More recent works [1,2,3,4] have emphasized the use of dual sinusoidal FOSs placement Figure 6 and Figure 7, which was shown to screen up some drawback of monitoring by a single sinusoidal FOS. Indeed, dual-sinusoidal placement offers maximum surface monitoring. More broadly, the most important point to be kept in mind is the wide potential of applications of distributed FOS in many structures and/or infrastructures, whatever the geometries, sizes, and shapes, provided that embedding is performed in a smart way that preserves the integrity and physical properties of sensors and parent structures as well. To allow these sensors to offer the full measure of their performances, without turning out to have “flaws” passing through their miniaturizing as much as possible, their placement far enough from the edges and implementing appropriate design and efficient technologies (right choice of fiber-optic cores, coatings, etc.) are the conditions that may guarantee the full and reliable use of the so-made smart structures.

In the following, the same reasoning conducted in the above for the case of a single high-voltage transmission phase is used. In the above, the monitoring has been optimized in order to ensure the maximum coverage of the area of the composite structure under surveillance, while in the case of the phase, the problem of helical copper wires is more complex, due to the multiplicity of constraints to which they are subjected and the fear of plasticity which threatens them.

## 6. Results and Analysis

A set of test cases were considered, trying to determine the best optimal location for embedding FOSs inside the core and/or as close as possible to the core. This is necessary because the closer the FOSs are to the core, the more accurate the monitoring results will be. Thus, FOSs were embedded close to the core but without compromising their safety. It is worthwhile to note that, here, the article is discussing the conceptualization of FOS embedding inside a single electric phase only, and it is still a little far from engineering applications, which would impose extensive experimental validation. Thus, the current article has to be considered mainly as a proof of concept.

(i) Test case A—Placement of linear FOS in parallel arrangement with varying length from the core.
(ii) Test case B—Placement of FOS helically wound around the complete core.

### 6.1. Test Case A—Placement of Linear FOS in Parallel Arrangement with Varying Length from the Core

**Figure 8:**
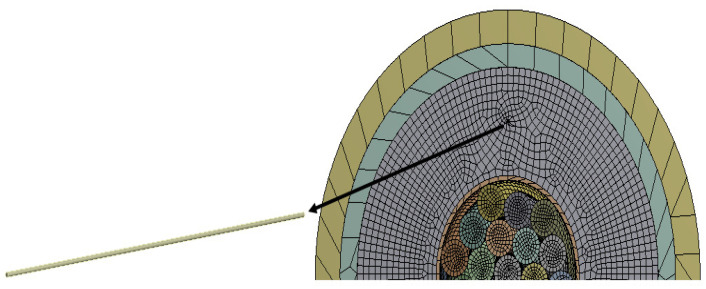
Placement of linear FOS 4 mm inside XLPE.

The longitudinal length of the linearly-placed FOS is considered in all simulations as 40 mm, which corresponds to the helical step. Figure 8 is one of the examples of the test case where linear FOS is embedded 4 mm inside the XLPE insulator. The strain values in various directions are represented by Exx in the figures.

**Figure 9:**
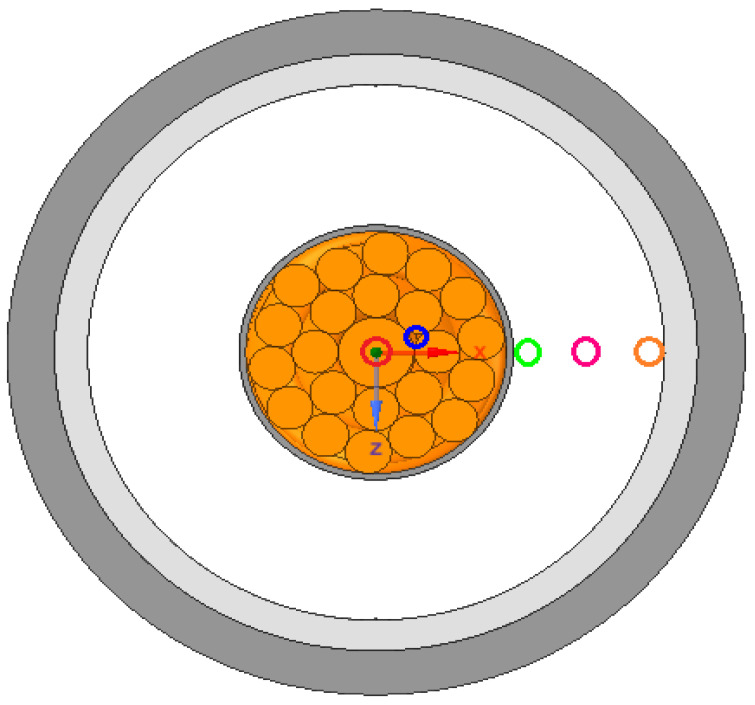
Color scheme for placement of FOS.

From Figure 9, different color schemes of linear-placed FOS can be observed. The blue color scheme refers to FOS placed parallel to the central wire, whereas the other color schemes of green, pink, and orange refer to the FOS embedded inside the XLPE insulator over the thickness. The length considered in all simulations is 40 mm, which corresponds to the helical pitch.

The FOS being in direct contact with copper wire is expected to record an averaged value of copper deformation. The red curve represent strain values of the central wire of the copper core. The strain values in the FOS which is placed parallel and in contact with the core (in blue) provides almost the same value of all directional strain as that of the copper wire (in red). Statistically, this seems to be the most convenient case which could be adopted, but arrangement of FOS parallel to the central wire of the core is risky and accounts for a high probability of failure, unless proper coating is used. All simulations were conducted using fiber-optic made of silica glass (density 2.4, Young’s modulus 72 GPa, and Poisson’s ratio 0.17) and acrylate coating (density 0.9, Young’s modulus 2.7 GPa, and Poisson’s ratio 0.35).

**Figure 10:**
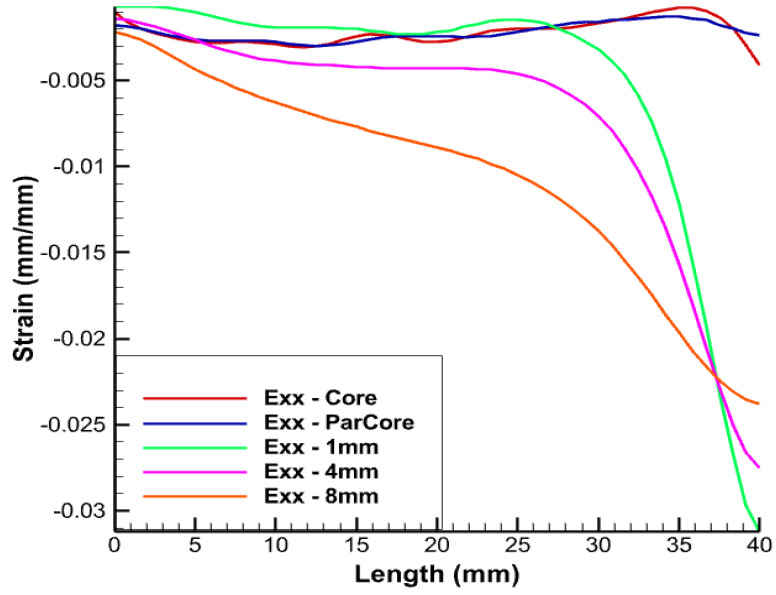
$E_{xx}$ strain for pure tension test case.

**Figure 11:**
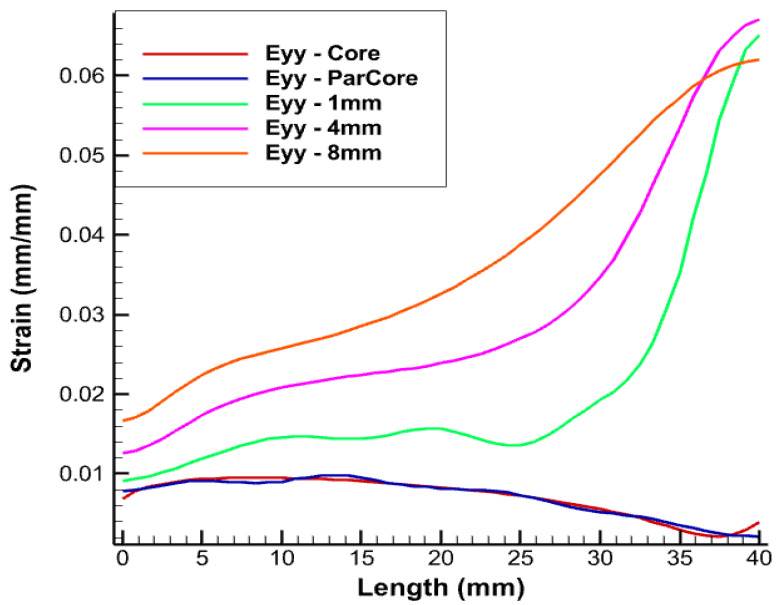
$E_{yy}$ strain for pure tension test case.

**Figure 12:**
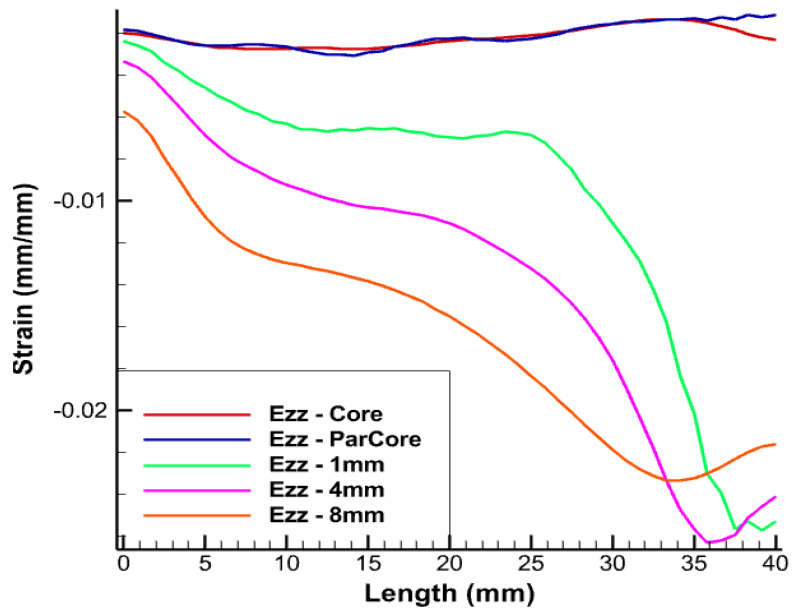
$E_{zz}$ strain for pure tension test case.

From Figure 10, Figure 11 and Figure 12, linear FOS placed 1 mm inside the XLPE insulator (in green) provides us with similar strain values to those of copper but it experiences significant divergence in strain values after a given FOS length. The most probable reason for this divergence is due to the boundary condition which is being applied at the free end. Another reason is that this placement corresponds to the case where the FOS sensor is embedded within two different media (copper and XPLE) that have different mechanical properties and, consequently, depending where mesh nodes are located, calculations are oscillating between two different values of the Young’s modulus, which may introduce some errors. This may come from numerical simulation.There are fluctuations in the calculation of the Young’s modulus that will randomly change depending on where the mesh noodles are located. Embedding FOS much further into the insulator, 4 mm inside the XLPE insulator (shown in pink) and away from the central core, was also tested. There is slight difference in the strain magnitude and there still exists divergence in the strain values after certain length of the FOS, which needs to be considered. This can also be considered as a potential case for the optimal placement of FOS. In the case of embedding FOS 8 mm inside the insulator, XLPE (shown in orange) nearly edges the boundary of the insulator screen.This lies approximately 15 mm away from the central core, which involves some offset in the values of the strain. From the figure, in spite of placing FOS at a comparatively far distance from copper core, similar trends and divergences exist when compared with the previous test case.

From the above results, a better perspective about the placement of FOS for accurate SHM can be observed. Placement of an FOS inside the copper core provides exactly the same results as that of copper, but technically implementing this case is somewhat risky and must be considered along with appropriate fiber-optics coating technologies. The same risk is still to be considered when FOS is helically wound the same way as copper wires, although this would bring the most valuable outputs, i.e., multi-parameter strains. Thus, this case can be considered if full assurance is given that FOS will not suffer failure following helical winding coupled to sliding/friction with copper wires. The second case with placement of FOS 1 mm inside the XLPE provides almost the same lateral strain ($E_{xx}$). There is not a major difference either in the longitudinal strain. With the other two cases, there exist a difference in longitudinal strain and lateral strain up to magnitude $e^{-2}$. The case with embedding FOS 1 mm inside the XLPE insulator (in green) is the most appropriate and reliable in terms of FOS safety and monitoring.

### 6.2. Test Case B: Placement of FOS Helically Wound around the Complete Core

**Figure 13:**
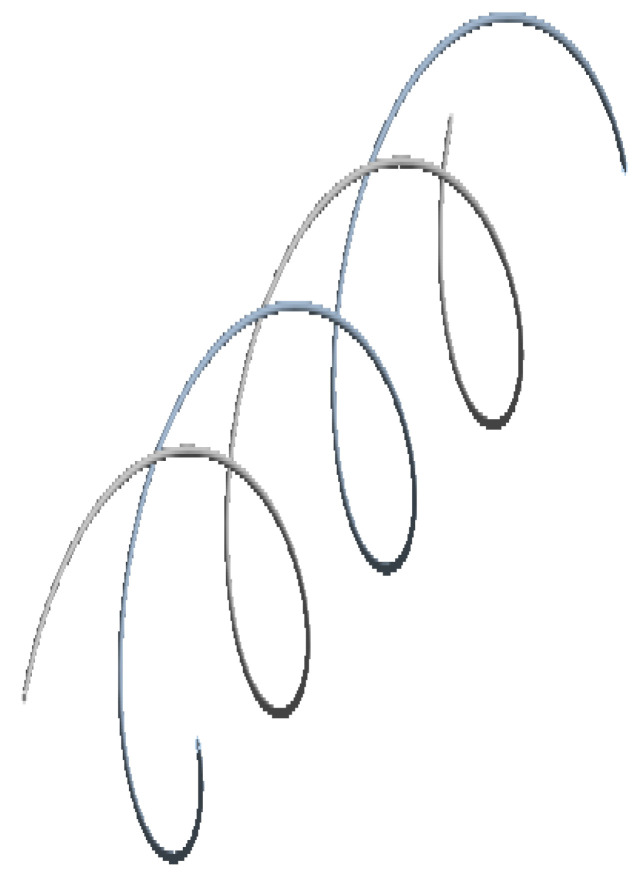
Helical arrangement FOS.

**Figure 14:**
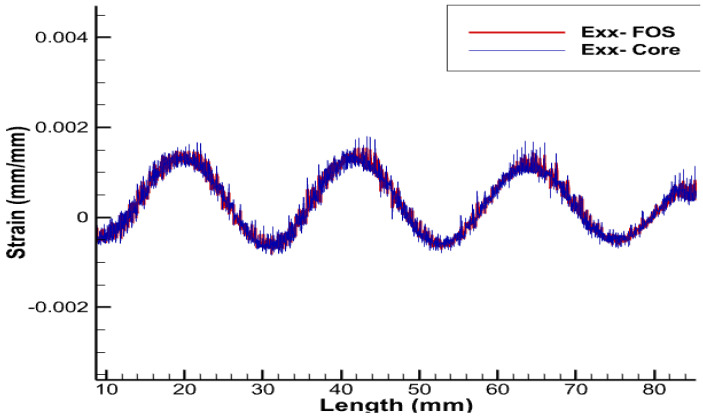
$E_{xx}$ strain for helical arrangement of FOS.

**Figure 15:**
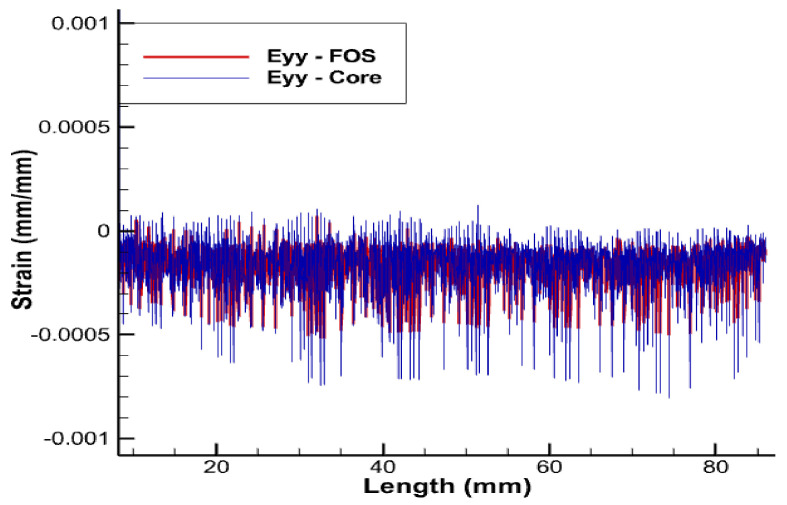
$E_{yy}$ strain for helical arrangement of FOS.

**Figure 16:**
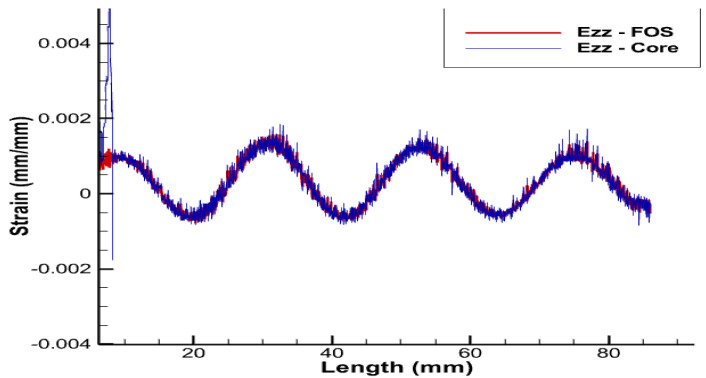
$E_{zz}$ strain for helical arrangement of FOS.

From previous research [3], using sinusoidal placement over the linear one provides multi-parametric strain values, i.e., strains in linear, shear, and lateral directions, whereas the linear FOS arrangement just provides the strain values in the lateral direction and is not sensible on longitudinal and shear loads [1]. Thus, FOS was also tested numerically, helically wound around a helical copper wire. The pitch size of the helical FOS is considered the same as that of the copper coil, i.e., pitch of the helical wire used is half of the total axial length. Along with this, the helical wound FOS covers a much greater area compared to parallel FOS arrangement [1]. This should tend to give much more complete results compared to the parallel FOS placement. In other words, one can hopefully access the complete information about the average status of partial and/or total plasticity of copper in the three dimensions, which is considerable, knowing that all the wires, except the central one, are helically-wound (Figure 13, Figure 14, Figure 15 and Figure 16).

Strain values of the outermost helical copper wire and the helical FOS were compared (Figure 13, Figure 14 and Figure 15). FOS is directly in contact with copper in this case, but the trend of both values of strains is almost the same. The issue with this case is the technical difficulty in placing the helically wound FOS around a single copper wires, knowing that helically-wound strategy is the most informative one since it wil come up with multiparameter strain monitoring of copper wires. Note that crumbling and twisting pressure force acting on the FOS from the core and from the conductor screen leaves a window for cause of failure. Also, during the process of installation and retrieval, there is also a high possibility of damaging the FOS when it brushes over the copper-wound wires.

This solution comes with a strong technology challenge, namely how to embed fiber-optic sensors between copper wires while ensuring they can withstand the stress–strain state of helically-wound copper wires. It is worthwhile to note that this is out of the scope of the current article for the time being. Our research should be viewed as no more than a preliminary numerical model that is trying to tackle a complex and challenging problem.

## 7. Conclusions and Discussion

The concept of using embedding FOSs inside high-voltage electric transport phases (not cables) for offshore farms was numerically introduced as a preliminary study to directly monitor true strain of copper wires. The idea is to be informed well before extensive plasticity develops within the wires, thus avoiding electric shutdown. The strain values of FOSs were compared with the strain values of copper cores. The test case where FOS was placed parallel to the central wire produces very good results, although integrity of FOS must be carefully checked by using appropriate coating technologies. The other test cases were favorable, of which the case of embedding the FOS 1 mm inside the XLPE insulator was promising, too. This could be further implemented and tested to perform the most reliable SHM for smart energy transport phases. The other test case, where the FOS is helically wound around and is directly in contact with copper core, provides the most information with the lateral, shear, and longitudinal strain all known at the same time, although it raises a strong technical challenge, which could be dealt with in the future upcoming developments. Finally, through this article, the option of embedding optical fiber within the phase for finer monitoring of plastic deformation of copper wires and their individual failure seems interesting, although extensive work pertaining to refining sensors placements, physics of sensors, and engineering still needs to be performed, to better emphasize the concept which is suggested. Current results, although not totally enough to validate the concept at the engineering step, do however light the way towards obtaining real, accurate, and in situ 3D strain of copper wires inside electric transport cables for offshore farms.

## Figures and Tables

**Table 1:** Material table assigned to single phase cable.

Materials	Density (kg/m${}^{3}$)	Young’s Modulus (MPa)	Poisson’s Ratio	Thermal Conductivity (W/mK)
Copper	8300	1.1 $\times \;10^{5}$	0.3	370
Semi-Conducting Polymer	1000	1500	0.4	0.1
XLPE	955	1250	0.4	0.28
Polyethelene Black	958	1050	0.4	0.2
Acrylate	950	2700	0.35	0.2
Polymide	1100	3000	0.42	0.8
